# Activation of the Ca^2+^-sensing receptors increases currents through inward rectifier K^+^ channels via activation of phosphatidylinositol 4-kinase

**DOI:** 10.1007/s00424-016-1901-y

**Published:** 2016-11-12

**Authors:** Chung-Hung Liu, Hsueh-Kai Chang, Sue-Ping Lee, Ru-Chi Shieh

**Affiliations:** 1 Institute of Biomedical Sciences, Academia Sinica, 128 Yen-Chiu Yuan Road, Section 2, 115 Taipei, Taiwan Republic of China; 2 Imaging Core, Institute of Molecular Biology, Academia Sinica, 115 Taipei, Taiwan Republic of China

**Keywords:** Calcium-sensing receptors, Inward rectifier K^+^ channels, PIP_2_, Membrane excitability

## Abstract

Inward rectifier K^+^ channels are important for maintaining normal electrical function in many cell types. The proper function of these channels requires the presence of membrane phosphoinositide 4,5-bisphosphate (PIP_2_). Stimulation of the Ca^2+^-sensing receptor CaR, a pleiotropic G protein-coupled receptor, activates both G_q/11_, which decreases PIP_2_, and phosphatidylinositol 4-kinase (PI-4-K), which, conversely, increases PIP_2_. How membrane PIP_2_ levels are regulated by CaR activation and whether these changes modulate inward rectifier K^+^ are unknown. In this study, we found that activation of CaR by the allosteric agonist, NPSR568, increased inward rectifier K^+^ current (*I*_K1_) in guinea pig ventricular myocytes and currents mediated by Kir2.1 channels exogenously expressed in HEK293T cells with a similar sensitivity. Moreover, using the fluorescent PIP_2_ reporter tubby-R332H-cYFP to monitor PIP_2_ levels, we found that CaR activation in HEK293T cells increased membrane PIP_2_ concentrations. Pharmacological studies showed that both phospholipase C (PLC) and PI-4-K are activated by CaR stimulation with the latter played a dominant role in regulating membrane PIP_2_ and, thus, Kir currents. These results provide the first direct evidence that CaR activation upregulates currents through inward rectifier K^+^ channels by accelerating PIP_2_ synthesis. The regulation of *I*_K1_ plays a critical role in the stability of the electrical properties of many excitable cells, including cardiac myocytes and neurons. Further, synthetic allosteric modulators that increase CaR activity have been used to treat hyperparathyroidism, and negative CaR modulators are of potential importance in the treatment of osteoporosis. Thus, our results provide further insight into the roles played by CaR in the cardiovascular system and are potentially valuable for heart disease treatment and drug safety.

## Introduction

Kir2.x channels are inward rectifier K^+^ channels that play an important role in maintaining stable resting membrane potentials, controlling excitability, and shaping the initial depolarization and final repolarization of ventricular myocytes [13, 19, 21, 27]. Gain and loss of function of Kir2.x channels, which mediate cardiac inwardly rectifying currents (*I*_K1_), can cause reentry and arrhythmia, respectively [19]. Reentry is facilitated by shortening of the action potential duration (APD), which abbreviates refractoriness. On the other hand, excessive APD prolongation may cause torsades de pointes arrhythmia and sudden cardiac death [25].

We previously demonstrated that extracellular spermine inhibits the outward current through Kir2.1 channels expressed in oocytes and the outward *I*_K1_ of myocytes, but the effect was much greater in oocytes than in cardiac myocytes [3]. However, why the effects of extracellular spermine are quantitatively different in oocytes and guinea pig myocytes is unclear [3]. It may be simply attributable to the fact that the effects of spermine on Kir channels in different cell types vary. Alternatively, the actions of extracellular spermine may be more diverse in complex cell types such as cardiac myocytes. For example, extracellular spermine can activate the calcium-sensing receptor, CaR [34]. This receptor was first discovered in the parathyroid gland, where its activation by extracellular Ca^2+^ was shown to decrease the release of parathyroid hormone [22]. CaR is also highly expressed in the kidney, bone, blood vessels, brain, and heart [34]. CaR is a pleiotropic G protein-coupled receptor and thus couples to more than one type of G protein [4]. Notably, several signaling pathways activated by stimulation of CaR regulate *I*_K1_. For example, increases in intracellular Ca^2+^ concentration ([Ca^2+^]_*i*_) and activation of protein kinase C (PKC) inhibit this current [5, 6, 15], whereas a rise in phosphoinositide 4,5-bisphosphate (PIP_2_) levels enhances the current [8]. PIP_2_, acting as a second messenger, plays an important role in modulating several ion channels and transporters [8, 30]. Stimulation of CaR activates both G_q/11_, which decreases PIP_2_, and phosphatidylinositol 4-kinase (PI-4-K), which increases PIP_2_. But how membrane PIP_2_ levels are regulated by CaR activation and whether the resulting changes regulate inward rectifier K^+^ channels are unknown.

In this study, we monitored PIP_2_ levels using the fluorescent PIP_2_ probe tubby-R332H-cYFP [12] and investigated how this regulation affects Kir2.1 channels expressed in HEK293T and *I*_K1_ in guinea pig ventricular myocytes.

## Materials and methods

### Cell culture and transfection procedures

HEK293T cells were cultured in Dulbecco’s modified Eagle’s medium (DMEM; Sigma Chemical Co., St. Louis, MO, USA) containing 10% (*v*/*v*) fetal bovine serum (FBS; Life Technologies, Paisley, Scotland) and 1% penicillin-streptomycin at 37 °C in a humidified atmosphere containing 5% CO_2_. HEK293T cells plated on poly-l-lysine-coated no. 1 glass coverslips (35 mm) were transiently transfected with 2 μg of the expression constructs Kir2.1-cyan fluorescent protein(eCFP), CaR-green fluorescent protein (GFP) (OriGene Technologies Inc., MD, USA), and/or tubby-R332H-yellow fluorescent protein (cYFP) using Lipofectamine 2000 (Invitrogen Co., Carlsbad, CA, USA). Cells were used 1–2 days after transfection. The Kir2.1-eCFP construct was generated by subcloning Kir2.1 cDNA into an *Xho*I/*Hin*dIII-digested peCFP vector (Clontech Lab Inc., Mountain View, CA, USA).

### Isolation of guinea pig cardiac myocytes

Guinea pig (Hartley) ventricular myocytes were isolated using an enzymatic procedure described previously [1]. Briefly, guinea pigs were anesthetized with sodium pentobarbital (50 mg/kg, i.v.) and hearts were isolated and retrogradely perfused, first with a Ca^2+^-free Tyrode’s solution (5 mM HEPES pH 7.4, 145 mM NaCl, 5 mM KCl, 2 mM MgCl_2_, 1 mM CaCl_2_, 5.5 mM glucose) and then with the same solution containing 0.5 mg/ml collagenase, 0.25 mg/ml protease, 1 mg/ml albumin, and 50 μM CaC1_2_. The heart was minced, and cells were dissociated by gentle agitation in the enzyme solution. Isolated cells were stored at room temperature in modified Tyrode’s solution (pH 7.4) containing 100 mg/ml albumin and 10 mM glucose.

### Electrophysiological recordings

Currents were recorded at room temperature (21–24 °C) using the patch-clamp technique [7] in the whole-cell configuration and an Axopatch 200B amplifier (Molecular Devices, Sunnyvale, CA, USA). The bath contained Tyrode’s solution (see composition previously mentioned), and the pipette solution (pH 7.2) contained 138 mM K-aspartate, 3 mM MgATP, 5 mM Na_2_-phosphocreatine, 1 mM MgCl_2_, 5 mM EGTA, 5 mM HEPES, and 2 mM creatine. For recordings of *I*_K1_ in myocytes, 5 μM nifedipine, 1 μM atropine, 10 μM glibenclamide, and 1 mM 4-aminopyridine were added in Tyrode’s solution.

Currents were sampled and filtered at frequencies of 20 and 5 kHz, respectively. Command voltages were controlled by and data were acquired using the pCLAMP10 software (Molecular Devices). Recordings in HEK293T cells and myocytes were corrected for the liquid junction potential (−15 mV).

### Measurement of fluorescence signals

Total internal reflection fluorescence (TIRF) fluorescence images of HEK293T cells were obtained at 22 °C using a Nikon Eclipse microscope with a Ti-E TIRF module (PFS) (Nikon Co, Tokyo, Japan) and an iXON Ultra 897 EMCCD camera (Andor Technology Ltd., Belfast, UK). Cells were visualized using a ×100 1.45 oil immersion objective lens. YFP fluorescence was monitored by exciting with a 515-nm laser (Cobolt Laser, Sweden) and collecting the emission at 535/30 nm (Chroma, Rockingham, VT). Averaged fluorescence intensity *F* of a region of interest was selected to cover the majority of the cell with background subtraction.

### Data analysis

Averaged data are presented as means ± SEMs. Student’s *t* test for independent samples was used to assess the statistical significance of differences. Asterisks *, **, and *** indicate *p* < 0.05, 0.01, and 0.005, respectively.

## Results

### Effects of CaR activation on the inhibition of Kir2.1 channels by extracellular spermine in HEK293T cells

**Fig. 1:**
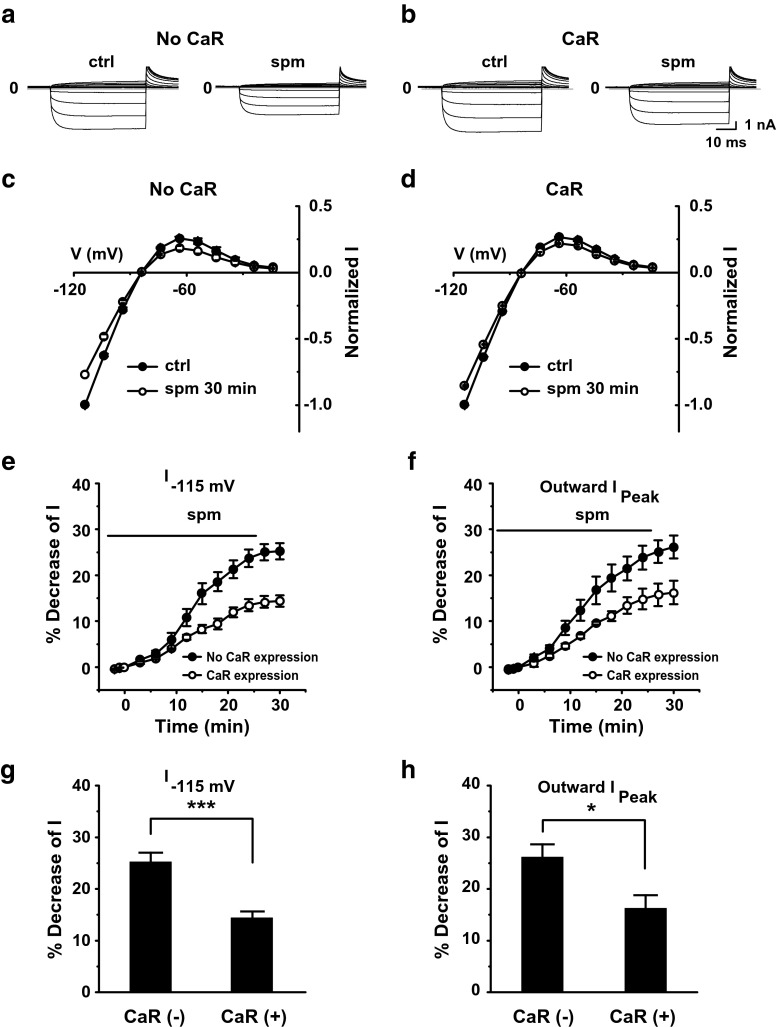
Effects of extracellular spermine on Kir2.1 currents. **a** Representative traces showing currents recorded using a voltage step protocol (−115 to −15 mV in 10-mV increments) from a holding potential of −15 mV in the whole-cell mode from a HEK293T cell transfected with Kir2.1 alone. **b** Currents recorded from a HEK293T cell transfected with Kir2.1 and CaR. **c**, **d** Effects of extracellular spermine on normalized I–V relationships in the absence and presence of CaR activation. Currents were normalized to that recorded at −115 mV under control condition. **e**, **f** Time course of spermine-inhibited currents, showing currents recorded at −115 mV (**e**) and peak outward currents (**f**) in the absence (*n* = 8) and presence (*n* = 6) of CaR expression. **g**, **h** Quantification of results in **e**, **f**, showing the percent decrease in the current recorded at −115 mV (**g**) and peak outward currents (**h**) in CaR-expressing cells compared with controls

To test whether spermine can indirectly modulate Kir2.1 channels through activation of CaR, we compared the effects of spermine in the absence and presence of exogenously expressed CaR. Currents were recorded from HEK293T cells transfected with Kir2.1 channels in the whole-cell configuration. Spermine decreased both inward and outward currents, but the magnitude of this decrease was diminished in the presence of CaR (Fig. 1a, b). The effects of spermine on the voltage dependence of normalized current (*I*) are shown in Fig. 1c, d. A comparison of the time course of the decrease in current recorded at −115 mV (Fig. 1e) and peak outward current (Fig. 1f) in the absence and presence of CaR expression showed that spermine-induced inhibition of currents was significantly reduced in HEK293T cells cotransfected with Kir2.1 and CaR compared with that in cells transfected with Kir2.1 alone (Fig. 1g, h). It is noted that the time course of effect was slow with and without CaR expression (Fig. 1e, f), suggesting that the direct effect of spermine on the Kir2.1 channel is as slow as the indirect effect (i.e., accumulation of PIP_2_ against the phospholipase C (PLC)-dependent hydrolysis via CaR activation). Our previous study suggests that extracellular spermine bound to the mouth of the extracellular pore of the Kir2.1 channel may induce an allosteric effect on voltage-dependent decay of outward currents, a process in which a region in the vicinity of the selectivity filter and cytoplasmic pore is involved [3]. The slow time course of effect by spermine may be due to this complicated allosteric regulation.

These results suggest that spermine can enhance Kir2.1 channel activity through activation of CaR in addition to its direct inhibitory effect on the channel.

### CaR activation increases currents mediated by Kir2.1 channels expressed in HEK293T cells

**Fig. 2:**
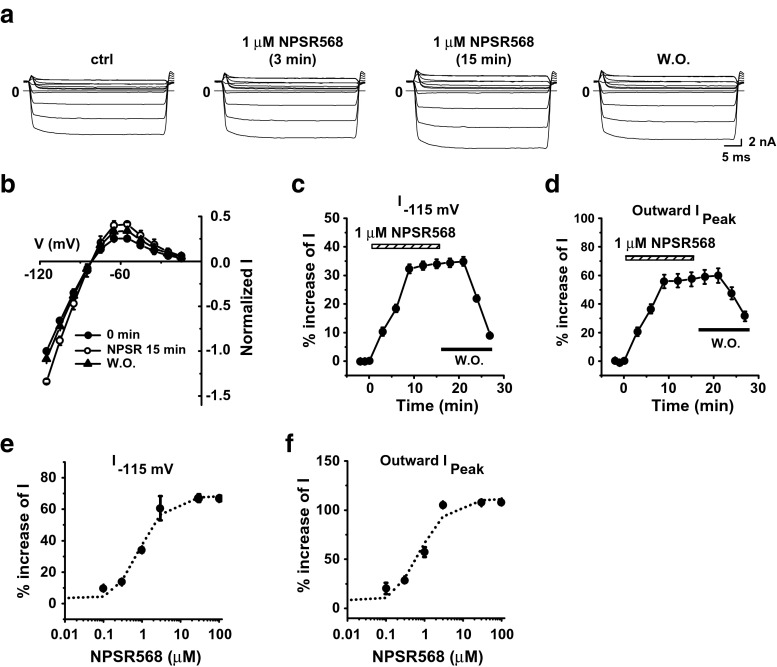
CaR stimulation increases Kir2.1 currents. **a** Effects of NPSR568 on currents recorded from a HEK293T cell transfected with both Kir2.1 and CaR. **b** Voltage dependence of normalized current. **c**, **d** Time courses of NPSR568 (1 μM)-induced averaged changes in currents, showing currents recorded at −115 mV (**c**) and peak outward currents (**d**), *n* = 6. **e**, **f** Concentration-response effects of NPSR568 on current (*n* = 3–6)

Next, we examined the effects of CaR activation alone on the Kir2.1 channel. Stimulation of CaR with 1 μM NPSR568 resulted in increases in both inward and outward currents at all voltages tested, and the effects were reversible upon washout (Fig. 2a, b). The time courses of the increases in current recorded at −115 mV and of peak outward current are shown in Fig. 2c, d, respectively. Fitting the concentration-response curve of NPSR568 to current recorded at −115 mV (Fig. 2e) and peak outward current (Fig. 2f) yielded *K*_a_ values of 0.86 μM at −115 mV and 0.71 μM for peak outward current. The maximum increase in current was 68.3% at −115 mV and 111.4% for peak outward current. These results support the conclusion that activation of CaR increases Kir2.1 currents.

### CaR activation increases Kir2.1 currents and PIP_2_ via activation of PI-4-K

**Fig. 3:**
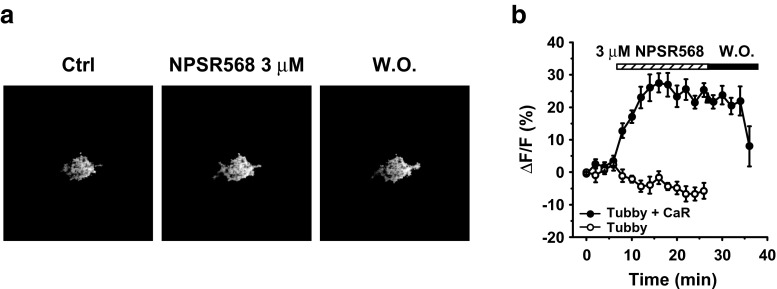
Activation of CaR increases membrane PIP_2_. **a** Images of tubby-R332H-cYFP fluorescence obtained from cells under control conditions, in the presence of 3 μM NPSR568 and following washout. **b** Time course of increases in fluorescence intensity at the membrane induced by 3 μM NPSR568, expressed as the percent change in fractional fluorescence (*n* = 13 for tubby-R332H-cYFP and CaR cotransfection; *n* = 11 for tubby alone)

CaR activation stimulates both the PLC pathway, which reduces membrane PIP_2_, and the PI-4-K pathway, which stimulates PIP_2_ synthesis. The enhancing effects of the CaR agonist, NPSR568, on Kir2.1 channel activity suggest that the predominant pathway is PIP_2_ synthesis. To test this hypothesis, we monitored PIP_2_ levels using a YFP-tagged, mutated C-domain of the PIP_2_-binding protein tubby (tubby-R332H-cYFP) [12, 24]. The application of 3 μM NPSR568 resulted in reversible increases in membrane fluorescence in cells expressing tubby-R332H-cYFP (Fig. 3a). The time courses of fluorescence changes at the membrane are shown in Fig. 3b. In cells expressing both tubby-R332H-cYFP and CaR, application of NPSR568 increased membrane fluorescence by 25.3 ± 2.1%. In cells transfected with tubby-R332H-cYFP alone, fluorescence was not increased upon treatment with NPSR568. The YFP fluorescence of cells transfected with CaR-GFP alone was too low to be detected and thus was not quantified.

**Fig. 4:**
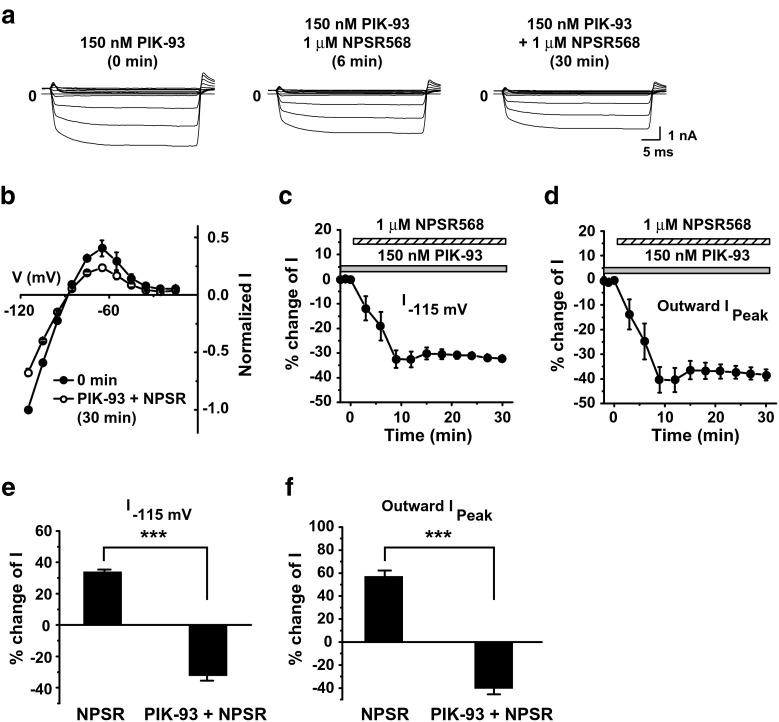
Effects of PIK-93 on NPSR568-induced increases in Kir2.1 currents. **a** Effects of NPSR568 with 150 nM PIK-93 pretreatment (10–15 min), on currents recorded from a HEK293T cell transfected with both Kir2.1 and CaR. **b** Voltage dependence of normalized I. **c**, **d** Time courses of NPSR568 (1 μM)-induced averaged changes in currents, showing currents recorded at −115 mV (**c**) and peak outward currents (**d**) with 150 nM PIK-93 pretreatment. **e**, **f** Quantification of results in **c**, **d**, showing the percent increase in currents recorded at −115 mV (**e**) and peak outward currents (**f**) induced by 1 μM NPSR568 alone (*n* = 6) and by 1 μM NPSR568 + 150 nM PIK-93 (*n* = 3)

The increase in membrane fluorescence induced by CaR activation implies that the PI-4-K pathway predominates over the PLC pathway in regulating Kir2.1 channels. To confirm this, we tested the effects of a potent inhibitor for PI-4-K IIIβ (which was attributed to membrane PIP_2_ increases upon CaR activation [11]), PIK-93 [2, 16], on CaR-induced increases in Kir2.1 currents and PIP_2_. With PIK-93 pretreatment (0.15 μM, 10–15 min), NPSR568 decreased both inward and outward currents (Fig. 4a, b), and this inhibitory effect increased over time (Fig. 4c, d). On average, with PIK-93 pretreatment, NPSR568 decreased currents at −115 mV and peak outward currents by 32.5 ± 3.2 and 40.5 ± 4.9%, respectively.

**Fig. 5:**
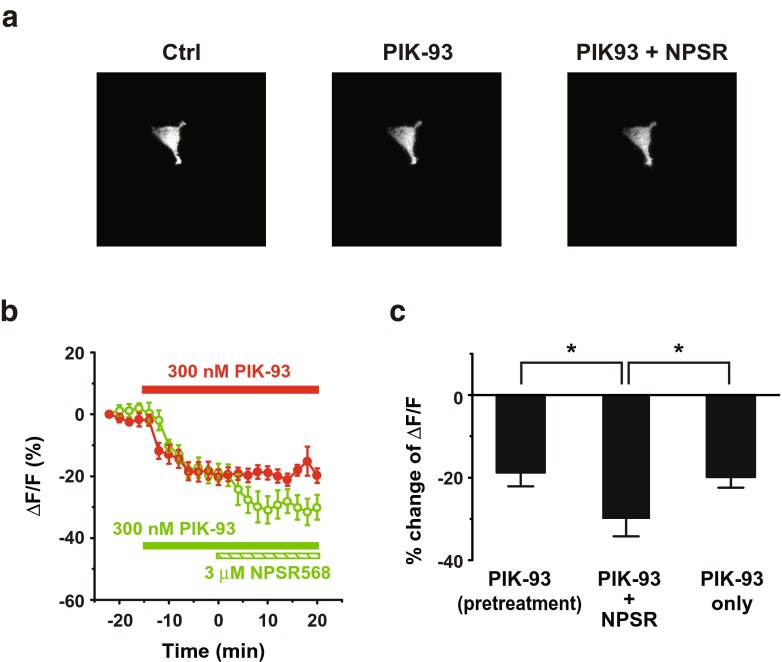
Effects of PIK-93 on NPSR568-induced increases in tubby-R332H-cYFP fluorescence on membrane. **a** Images of tubby-R332H-cYFP fluorescence obtained from cells under control (*left panel*), during PIK-93 pretreatment (*middle panel*), and after 3-μM NPSR568 application with 300 nM PIK-93 (*right panel*). **b** Time course of averaged changes in tubby-R332H-cYFP fluorescence at the membrane induced by treatment with 3 μM NPSR568 + 300 nM PIK-93 and PIK-93 alone. **c** Quantification of results in **b**, showing the percent increase in fractional fluorescence of tubby-R332H-cYFP at the membrane induced by treatment with 3 μM NPSR568 alone versus 3 μM NPSR568 + 300 nM PIK-93 (*n* = 11 for both sets of experiments)

Next, we examined the effects of PIK-93 on PIP_2_ levels. PIK-93 decreased membrane fluorescence, and the subsequent activation of CaR with 3 μM NPSR568 further reduced the signal (Fig. 5a). The time courses of fluorescence changes at the membrane are shown in Fig. 5b. PIK-93 treatment decreased the fluorescence by 18.8 ± 2.9%, and subsequently, application of NPSR568 further significantly decreased the signal to −30.1 ± 4.0% (Fig. 5c). PIK-93 treatment alone decreased the fluorescence by 19.8 ± 2.9%.

**Fig. 6:**
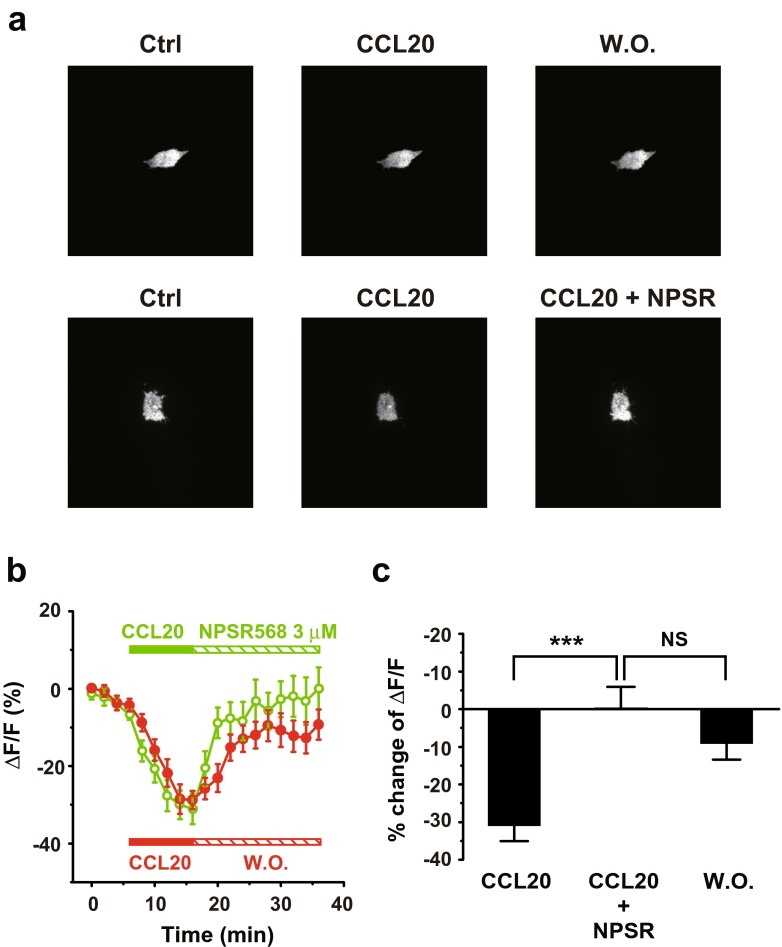
Stimulation of CaR promotes tubby-R332H-cYFP fluorescence recovery from PLC activation. **a** Images of tubby-R332H-cYFP fluorescence obtained from cells with CCR6, tubby, and CaR expression. The cell was first treated with CCL20 (300 ng/ml, *middle panel*) followed by washout (*right panel*). **b** Images of tubby-R332H-cYFP fluorescence obtained from cells first treated with CCR20 (*middle panel*) followed by application of 3-μM NPSR568 application (*right panel*). **c** Averaged percent of changes in fractional fluorescence of tubby-R332H-cYFP at the membrane induced with (*n* = 14) and without NPSR (*n* = 12)

To further support the involvement of PI-4-K in the enhancement of membrane (PIP_2_) induced by CaR activation, we next examined the recovery of membrane PIP_2_ level following PLC activation. Applying CCL20 (300 ng/ml) activated PLC via CCR6 [28] in stable transfected HEK293 cells and resulted in decreases of membrane fluorescence (Fig. 6a). Applying NPSR568 facilitated the recovery of tubby-R332H-cYFP fluorescence as compared to no NPSR treatment (Fig. 6b). Stimulation of CaR facilitated fluorescence recovery (*τ* = 3.7 min) as compared to the washout (*τ* = 5.8 min). CaR activation increased membrane fluorescence from −31.0 ± 4.0 to 0.1 ± 5.5%, and washout increased the signal to −9.3 ± 3.9% (Fig. 6c). These results support the conclusion that activation of CaR increases Kir2.1 channel-mediated currents by increasing the membrane PIP_2_ levels through activation of PI-4-K.

### CaR signaling through the G_q/11_ pathway inhibits Kir2.1 channel activity

**Fig. 7:**
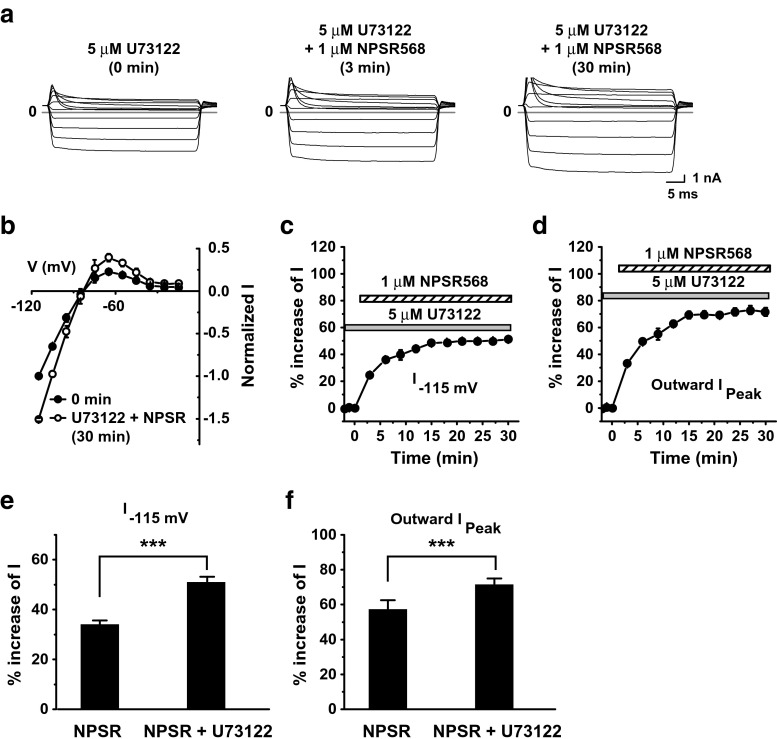
Effects of U73122 pretreatment on NPSR568-induced increases in Kir2.1 currents. **a** Effects of NPSR568 with 5-μM U73122 pretreatment (10–15 min) on currents recorded from a HEK293T cell transfected with both Kir2.1 and CaR. **b** Voltage dependence of normalized currents. **c**, **d** Time courses of NPSR568 (1 μM)-induced changes in currents, showing currents recorded at −115 mV (**c**) and peak outward currents (**d**), with 5-μM U73122 pretreatment. **e**, **f** Quantification of results in **c**, **d**, showing the percent change in currents recorded at −115 mV (**e**) and peak outward currents (**f**) induced by 1 μM NPSR568 alone (*n* = 6) and 1 μM NPSR568 plus 5 μM U73122 (*n* = 4)

In addition to activating PI-4-K, CaR stimulation also activates PLC via the G_q/11_ pathway and thus degrades membrane PIP_2_. To examine whether this pathway is involved in the regulation of Kir2.1 channels by CaR activation, we tested the effects of pretreatment of the PLC inhibitor, U73122, on CaR activation-induced increases in Kir2.1 currents and PIP_2_. With U73122 pretreatment (10–15 min), NPSR568 increased currents (Fig. 7a, b), and this effect increased over time (Fig. 7c, d). U73122 pretreatment significantly increased the effects of NPSR 568 on inward currents at −115 mV from 34.1 ± 1.6 to 51.1 ± 2.0 and peak outward currents from 57.3 ± 4.9 to 71.6 ± 3.0 (Fig. 7e, f).

**Fig. 8:**
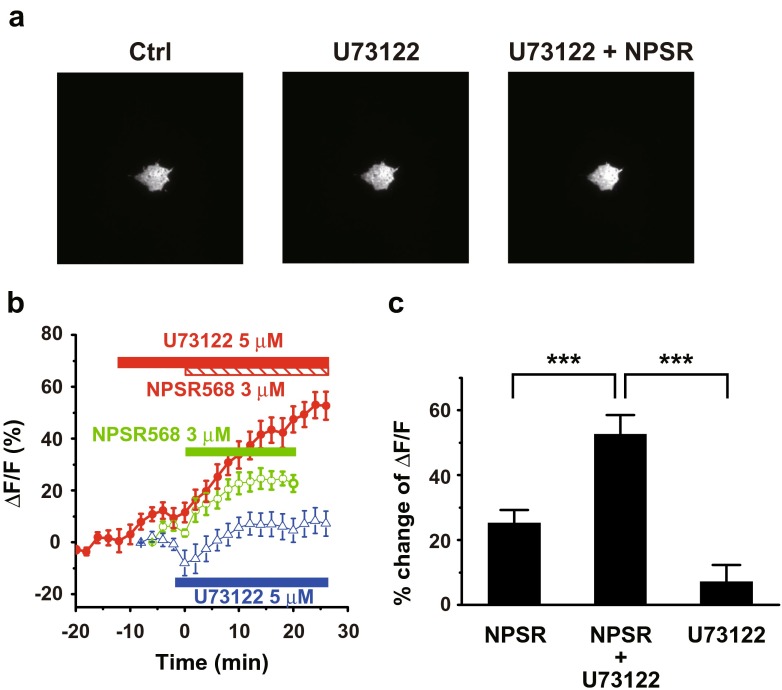
Effects of U73122 on NPSR568-induced increases in tubby-R332H-cYFP fluorescence on membrane. **a** Images of tubby-R332H-cYFP fluorescence obtained from cells under control (*left panel*), during U73122 pretreatment (*middle panel*), and after (right panel) of 3-μM NPSR568 application with 5 μM U73122 presence all the time. **b** Time course of averaged changes in tubby-R332H-cYFP fluorescence at the membrane induced by treatment with 3 μM NPSR568, 3 μM NPSR568 + 5 μM U73122, and 5 μM U73122 alone. **c** Averaged percent increase in fractional fluorescence of tubby-R332H-cYFP at the membrane induced by NPSR568 with and without U73122 pretreatment (*n* = 12) and by U73122 alone (*n* = 11)

Next, we examined the effects of U73122 pretreatment on PIP_2_ levels. With pretreatment of U73122, NPSR568 increased membrane tubby-R332H-cYFP fluorescence (Fig. 8a, right panel). The time courses of fluorescence changes at the membrane are shown in Fig. 8b. In cells treated with NPSR568 only, tubby-R332H-cYFP fluorescence was increased by 25.3 ± 2.1%. This effect was significantly enhanced by U73122 pretreatment to 52.7 ± 5.4% (Fig. 8c). The treatment of U73122 alone increased the fluorescence by 7.7 ± 4.8%, significantly lower than the treatment of U73122 + NPSR568 (Fig. 8c).

These results suggest that activation of CaR decreases Kir2.1 channel-mediated currents through activation of PLC.

### CaR activation increases *I*_K1_ in guinea pig ventricular myocytes

**Fig. 9:**
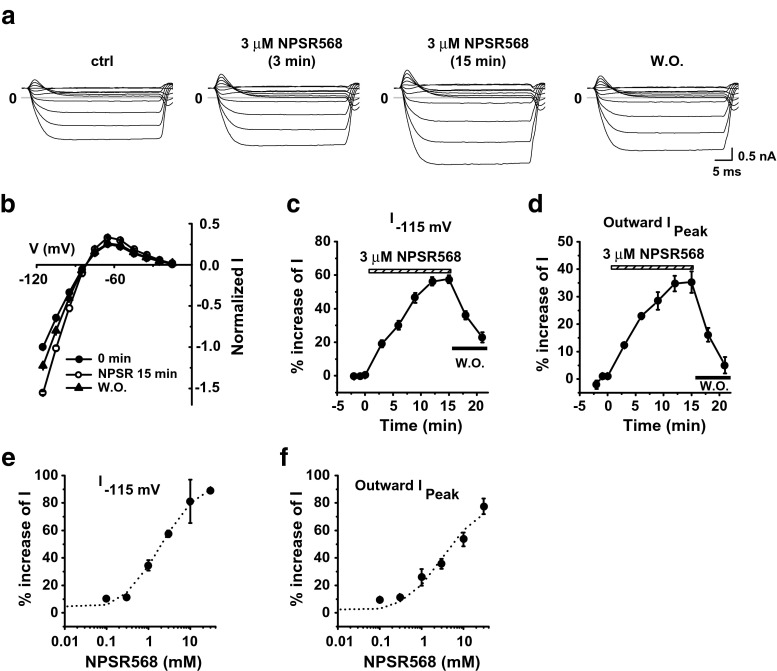
CaR stimulation increases *I*_K1_ in guinea pig ventricular myocytes. **a** Effects of NPSR568 on *I*_K1_ recorded from a guinea pig ventricular myocyte. **b** Voltage dependence of normalized *I*_K1_. **c**, **d** Time courses of averaged changes in *I*_K1_ induced by 3 μM NPSR, showing currents recorded at −115 mV (**c**) and peak outward currents (**d**), *n* = 6. **e**, **f** Concentration-response relationship, showing *I*_K1_ recorded at −115 mV (**e**) and peak outward *I*_K1_ (**f**) as a function of NPSR568 concentration (*n* = 3–6)

Next, we explored whether activating CaR regulates *I*_K1_ in native ventricular myocytes exposed to physiological solutions. Activation of endogenous CaR in ventricular myocytes with 3 μM NPSR568 resulted in increases in both inward and outward currents, and these effects were reversible upon washout (Fig. 9a, b). The time courses of averaged increases in currents recorded at −115 mV and in peak outward current are shown in Fig. 9c, d, respectively. An analysis of the concentration-response effect of NPSR568 on currents recorded at −115 mV (Fig. 9e) and on peak outward current (Fig. 9f) yielded *K*_a_ values of 1.82 and 3.22 μM, respectively. The maximum increase in current was 96.8% at −115 mV and 81.1% for peak outward current. These results support the conclusion that activation of CaR increases *I*_K1_ in guinea pig ventricular myocytes.

**Fig. 10:**
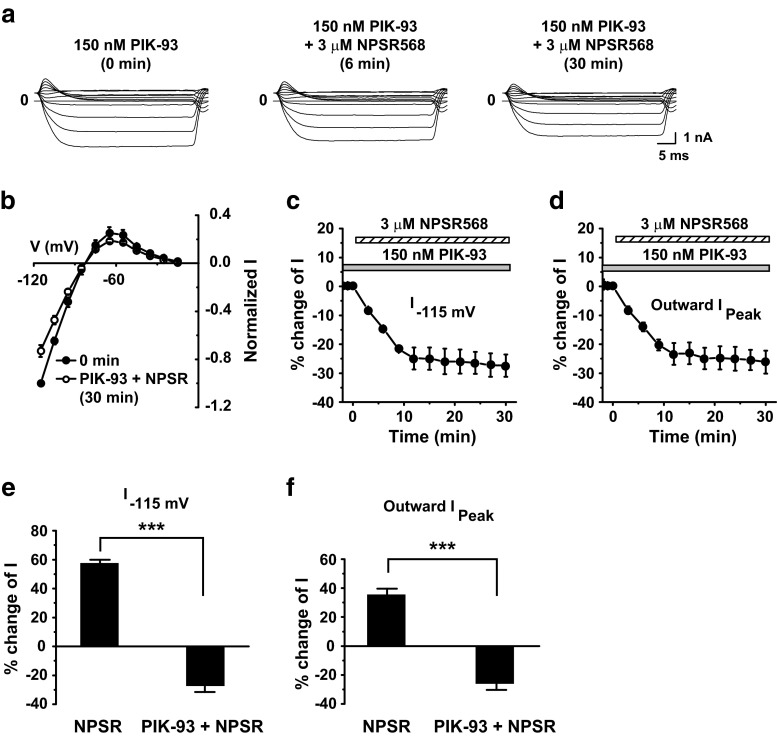
Effects of PIK-93 on NPSR568-induced increases in *I*_K1_. **a** Effects of NPSR568 with 150 nM PIK-93 pretreatment (10–15 min) on *I*_K1_ recorded from a guinea pig ventricular myocyte. **b** Voltage dependence of normalized *I*_K1_. **c**, **d** Time courses of averaged changes in *I*_K1_ induced by 3 μM NPSR, showing currents recorded at −115 mV (**c**) and peak outward currents (**d**) with 150 nM PIK-93 pretreatment. **e**, **f** Quantification of results in **c**, **d**, showing the percent change in currents recorded at −115 mV (**e**) and peak outward currents (**f**) induced by 3 μM NPSR568 alone (*n* = 6) and by 3 μM NPSR568 plus 150 nM PIK-93 pretreatment (*n* = 4)

Figure 4 shows that the enhancing effect of the CaR agonist NPSR568 on Kir2.1 channel activity was attributable to PIP_2_ synthesis through activation of PI-4-K. To confirm this mechanism in guinea pig ventricular myocytes, we tested the effect of PIK-93 pretreatment on CaR activation-induced increases in *I*_K1_. With PIK-93 pretreatment (0.15 μM, 10–15 min), 3 μM NPSR 568 decreased both inward and outward *I*_K1_ (Fig. 10a, b), and this inhibitory effect increased over time (Fig. 10c, d). With PIK-93 (0.15 μM) pretreatment, CaR activation decreased currents at −115 mV by 27.4 ± 3.9% and peak outward currents by 26.2 ± 4.0%.

**Fig. 11:**
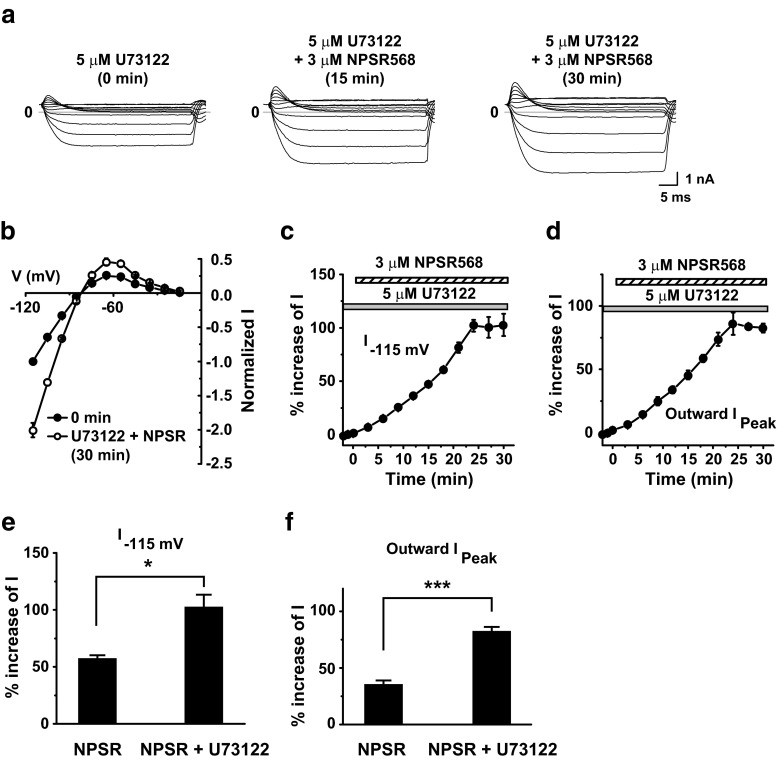
Effects of U73122 pretreatment on NPSR568-induced increases in *I*_K1_. **a** Effects of NPSR568 on *I*_K1_, with 5-μM U73122 pretreatment (10–15 min). **b** Voltage dependence of normalized *I*_K1_. **c**, **d** Time course of averaged changes in *I*_K1_ induced by 3 μM NPSR, showing currents recorded at −115 mV (**c**) and peak outward currents (**d**) with 5-μM U73122 pretreatment. **e**, **f** Quantification of results in **c**, **d**, showing the percent change in currents recorded at −115 mV (**e**) and peak outward currents (**f**) induced by 3 μM NPSR568 alone (*n* = 6) and by 3 μM NPSR568 plus 5 μM U73122 (*n* = 5)

Finally, to determine whether activation of PLC via the G_q/11_ pathway is involved in the regulation of *I*_K1_ by CaR activation in guinea pig ventricular myocytes, we tested the effect of U73122 pretreatment on CaR activation-induced increases in *I*_K1_. With U73122 pretreatment (5 μM, 10–15 min), NPSR 568 increased inward and outward *I*_K1_ (Fig. 11a–d). U73122 pretreatment significantly enhanced the stimulatory effect of NPSR568 on *I*_K1_ from 57.6 ± 2.4 to 102.7 ± 10.4% at −115 mV and from 35.7 ± 3.6 to 82.6 ± 3.5% for peak outward *I*_K1_ (Fig. 11e, f).

In summary, CaR stimulation activates both the PLC and PI-4-K pathways, but the effect on PI-4-K dominates, resulting in increases of Kir2.1 currents recorded from HEK293T cells transfected with Kir2.1 and *I*_K1_ in guinea pig ventricular myocytes.

## Discussion

### CaR activation increases membrane PIP_2_ levels and Kir currents

The purpose of this study was to examine whether CaR activation regulates inward rectifier K^+^ channels by modulating the level of membrane PIP_2_. To achieve this goal, we investigated the effects of activating CaR on currents mediated by Kir2.1 channels expressed in HEK293T cells and on *I*_K1_ in guinea pig ventricular myocytes. We found that activation of CaR by an allosteric agonist (NPSR568) increased both endogenous *I*_K1_ and currents mediated by exogenously expressed Kir2.1 to a similar extent. Further, monitoring PIP_2_ levels using the PIP_2_-binding probe tubby-R332H-cYFP demonstrated that CaR activation increased PIP_2_ concentration at the plasma membrane in HEK293T cells. These effects were abolished by PIK-93, suggesting the involvement of PI-4-K. CaR activation facilitates the recovery of membrane PIP_2_ following PLC activation, further supporting this notion. Collectively, these results provide the first direct evidence that CaR activation increases inward rectifier K^+^ channel currents by accelerating PIP_2_ synthesis in HEK293T cells and possibly also in guinea pig ventricular myocytes.

PIP_2_ acts as a second messenger molecule to play an important role in modulating a number of ion channels and transporters [8, 9, 30]. Previous literature reports have enhanced our understanding of this topic, yet significant gaps remain. First, although it has been shown that CaR can regulate ion channels in cells [33], CaR regulation of ion channels through modulation of PIP_2_ is a mechanism that has not been explored. Second, most previous investigations have focused on single signaling pathways in PIP_2_ regulation and its association with channel activities. Yet, multiple pathways regulate membrane PIP_2_, and how they are integrated to regulate ion channels remains elusive. In the current study, we explored the involvement of two signaling pathways engaged by activation of CaR in the regulation of PIP_2_. Our data revealed that activation of CaR results in increases in membrane PIP_2_ and Kir currents through PI-4-K activation, although the activation of PLC also makes contribution to the regulation of Kir channels.

Third, most studies have focused on the effect of PIP_2_ depletion on ion channels expressed in heterologous systems [23, 24, 30]. PIP_2_ depletion has also been studied in native cells. For example, it has been shown that, in normal isolated Müller cells, activation of G_q/11_-coupled metabotropic glutamate receptors (mGluRs) with the mGluR I agonist (S)-3,5-dihydroxyphenylglycine (DHPG) suppresses Kir currents through the intracellular Ca^2+^-dependent PLC/IP_3_-ryanodine/PKC signaling pathway [14]. On the other hand, little is known about whether resynthesis of PIP_2_ regulates ion channels in heterologous expression systems or in native cells. PI-4-K is involved in the constitutive biosynthesis of PIP_2_ and in PIP_2_ resynthesis after its breakdown by PLC. Can physiological regulators that target PI-4-K affect PIP_2_ levels and modulate channel activities? A previous study has shown that resynthesis of PIP_2_ via PI-4-K mediates adaptation of caffeine responses in taste receptor cells by regulating Kir and K_V_ channels [38]. Our study provides additional evidence that increasing membrane PIP_2_ by stimulating PI-4-K via CaR activation can regulate ion channel function.

Fourth, it is unclear whether physiological fluctuations in the levels of PIP_2_, as may occur with the activation of intracellular signaling pathways, are sufficient in and of themselves to regulate ion channels [9]. It has been hypothesized that some channels interact with lipid partners with high affinity such that the lipid-binding site of the channel remains saturated during most physiological changes in lipid levels. By contrast, other channels bind with low affinity; for these channels, variations in lipid concentration may act as physiological signals to regulate the computational properties of neurons and transport properties of secretory cells [23]. Among Kir channels, the constitutively active Kir1.1 and Kir2.1 interact with PIP_2_ with high affinity [10], whereas Kir3 channels show weaker interactions with PIP_2_ [26]. Application of exogenous PIP_2_ dynamically activates these channels in reverse order, namely Kir3.1/3.4 > Kir2.1 > Kir1.1 [10, 26, 36]. Whether Kir2.1 channels are sensitive to changes in membrane PIP_2_ level under physiological conditions remains unclear. Our study provides the first direct evidence that activation of CaR can increase *I*_K1_ via the PI-4-K pathway.

The modulation of Kir2.1 channels by PIP_2_ via CaR activation appears to be voltage dependent. The underlying mechanisms are unknown. It has been previously shown that polyamines bound to the Kir2.1 channel at positive driving force interact with the gating of Kir2.1 channels by PIP_2_ [35]. It is possible that the voltage-dependent effect of PIP_2_ is related to the voltage-dependent polyamine block of the channel. Further, it is shown that PIP_2_ shifts the voltage dependence of conductance by promoting the opening of the ion conduction gate and by a negative surface charge in Slo1 BK channels [32]. PIP_2_ may also exert similar effects on Kir, and thus, the effect appears to be different at different voltages.

In addition to regulating PIP_2_, CaR also modulates several cellular events including increases of [Ca^2+^]_*i*_, PKC, and arachidonic acids as well as a decrease in PKA [20, 33]. Several of these intracellular signaling molecules regulate Kir2.1 and/or *I*_K1_ [5, 17, 18, 40]. This study investigated only the effect of PIP_2_ on Kir2.1 upon CaR activation. How other intracellular signaling is involved in the regulation of Kir2.1/*I*_K1_ via CaR activation requires further studies. Further, besides *I*_K1_, the electrical properties of the membrane involve other ion channels. The overall physiological and pathological functions require further investigation when all the effects during CaR activation are taken into consideration.

In this study, we used PIK-93 to inhibit PI-4-K IIIβ. Because PIK-93 inhibits PI-3-K and PI-4-K with similar potency [2, 16], the contribution of PI-4-K-induced increases of Kir2.1 currents and *I*_K1_ upon CaR activation is underestimated. Our study provides the qualitative analysis of involvement of PI-4-K signaling in CaR activation. For more quantitative analysis, future studies with specific PI-4-K inhibitors are required.

### Pathophysiological implications of CaR regulation of *I*_K1_

It has been previously shown that putrescine significantly reduces the arrhythmia associated with cardiac ischemia and reperfusion [31]. Furthermore, ischemia/reperfusion results in depletion of the myocardial polyamine pool. Application of exogenous spermine restores the intracellular polyamine pool and reduces cardiac myocyte necrosis, suggesting that the loss of spermine might be involved in the cardiac injury produced by reperfusion [39]. These cardioprotective effects have been attributed to the membrane-stabilizing and antioxidative effects of putrescine [31]. However, because ischemia and reperfusion can lead to upregulation of CaR in the heart during a myocardial infarction [37] and because polyamines are potent agonists of CaR, it is possible that the cardioprotective effects of polyamines are the result of CaR activation. The results from our study suggest that CaR activation may stabilize the electrical properties of the cardiac membrane by increasing *I*_K1_. This action may play an important role in the cardioprotective effects of polyamines during ischemia/reperfusion injury, although further studies are required to confirm this hypothesis. CaR functions as an integrator of extracellular stimuli and is probably always activated to some extent [29]. It remains to be determined whether CaR calcilytics are capable of decreasing *I*_K1_ and thus affect cardiac electrical properties.

Extracellular Ca^2+^ is a low-affinity agonist for CaR, and thus, under physiological conditions, CaR is subjected to the tonic stimulation of extracellular Ca^2+^. Investigations on this tonic activation of CaR may shed light on the physiological functions of CaR.

### Conclusions

Our data show that increases of endogenous PIP_2_ in cardiac myocytes regulate *I*_K1_. This novel relationship supports a model in which autonomic stimulation of cardiac CaR may alter *I*_K1_-dependent repolarization and excitability and impact cardiac function. Our study explored important questions related to PIP_2_ modulation by CaR activation and showed that CaR can upregulate inward rectifier K^+^ channels in both native cells and a heterologous expression system. Given the increasing clinical use of calcimimetics in the treatment of hyperparathyroidism and the potential for using calcilytics in the treatment of osteoporosis, it is essential to understand the role of CaR in cardiac function [29].
